# Molecular Biology Networks and Key Gene Regulators for Inflammatory Biomarkers Shared by Breast Cancer Development: Multi-Omics Systems Analysis

**DOI:** 10.3390/biom11091379

**Published:** 2021-09-18

**Authors:** Su Yon Jung, Jeanette C. Papp, Matteo Pellegrini, Herbert Yu, Eric M. Sobel

**Affiliations:** 1 Translational Sciences Section, School of Nursing, University of California, Los Angeles, CA 90095, USA; 2 Jonsson Comprehensive Cancer Center, University of California, Los Angeles, CA 90095, USA; jcpapp@ucla.edu; 3 Department of Human Genetics, David Geffen School of Medicine, University of California, Los Angeles, CA 90095, USA; esobel@ucla.edu; 4 Department of Molecular, Cell and Developmental Biology, Life Sciences Division, University of California, Los Angeles, CA 90095, USA; matteop@mcdb.ucla.edu; 5 Cancer Epidemiology Program, University of Hawaii Cancer Center, Honolulu, HI 96813, USA; HYu@cc.hawaii.edu; 6 Department of Computational Medicine, David Geffen School of Medicine, University of California, Los Angeles, CA 90095, USA

**Keywords:** CRP/IL6, breast cancer, multi-omics integration, system biology, molecular pathways, gene network, key drivers

## Abstract

As key inflammatory biomarkers C-reactive protein (CRP) and interleukin-6 (IL6) play an important role in the pathogenesis of non-inflammatory diseases, including specific cancers, such as breast cancer (BC). Previous genome-wide association studies (GWASs) have neither explained the large proportion of genetic heritability nor provided comprehensive understanding of the underlying regulatory mechanisms. We adopted an integrative genomic network approach by incorporating our previous GWAS data for CRP and IL6 with multi-omics datasets, such as whole-blood expression quantitative loci, molecular biologic pathways, and gene regulatory networks to capture the full range of genetic functionalities associated with CRP/IL6 and tissue-specific key drivers (KDs) in gene subnetworks. We applied another systematic genomics approach for BC development to detect shared gene sets in enriched subnetworks across BC and CRP/IL6. We detected the topmost significant common pathways across CRP/IL6 (e.g., immune regulatory; chemokines and their receptors; interferon γ, JAK-STAT, and ERBB4 signaling), several of which overlapped with BC pathways. Further, in gene–gene interaction networks enriched by those topmost pathways, we identified KDs—both well-established (e.g., JAK1/2/3, STAT3) and novel (e.g., CXCR3, CD3D, CD3G, STAT6)—in a tissue-specific manner, for mechanisms shared in regulating CRP/IL6 and BC risk. Our study may provide robust, comprehensive insights into the mechanisms of CRP/IL6 regulation and highlight potential novel genetic targets as preventive and therapeutic strategies for associated disorders, such as BC.

## 1. Introduction

Chronic inflammation plays an important role in the pathogenesis of non-inflammatory diseases, including metabolic syndrome [1,2] and specific types of cancers, such as colorectal, liver, and breast cancers [3,4]. In particular, for carcinogenesis and cancer progression, C-reactive protein (CRP) and interleukin-6 (IL6) are key cancer-promoting inflammatory cytokines that are interrelated in oncogenesis and tumor growth through different molecular pathways in response to acute and chronic inflammation [4]. In detail, innate immune activation promotes the production of such pro-inflammatory markers, creating a tissue-specific microenvironment high in reactive oxygen and nitrogen species, resulting in DNA damage and alterations in nearby cells [5,6,7]. Specifically, the two cytokines have yielded a congruent association with the risk of breast cancer (BC) [8,9,10,11], although their carcinogenetic mechanisms are not fully understood.

Systemic development of those inflammatory markers can be influenced by not only environmental [12,13,14] but also genetic and epigenetic factors [15,16]. Despite advances in the understanding of genetic variance and gene–environment (G × E) interactions in relation to those biomarkers, common genetic variants from genome-wide association studies (GWASs) explain a small proportion of inter-individual variability (CRP < 5%; IL-6 < 2%) [17,18], indicating that a large proportion of heritability is undetermined.

Conventional GWASs cannot easily address several crucial issues. For example, a genomic study at the genome-wide level examines single genetic markers one at a time, revealing a small number of top genetic variants which explain a limited proportion of genetic heritability due to severe multiple testing corrections, suggesting the importance of group-level approaches in genomic studies [19]. Also, GWASs may not investigate tissue-specific gene–gene (G × G) interactions, which have received growing attention as a possible source for the missing heritability. Further, they cannot address functional characterization of the genetic variants/loci; thus, the molecular mechanisms by which the genetic perturbations influence the tested phenotype in downstream signaling cascades, further being involved in the complex process of related-disease development, are not elucidated.

Many pathway- and network-based approaches integrating GWAS findings with genetic and genomic expression data have demonstrated a powerful ability to detect the missing heritability of quantitative phenotypes and to unravel the genomic functionality on the basis of enriched molecular signaling cascades and genomic involvement in an associated-disease molecular process [20,21,22,23,24,25]. Further, gene regulatory network analysis in a tissue-specific manner can capture causal regulatory relationships between genes by accounting for G × G interactions in different pathologic conditions and identify key driver (KD) genes as important regulators of the particular enriched pathways.

For these reasons, we adopted an integrative genomic network approach (Figure S1) by incorporating our previous GWAS data for CRP and IL6 [26] with functional genomics data, including whole-blood expression quantitative loci (eQTLs, which capture functional regulation of gene expression); molecular biologic pathways; and G × G interaction information from data-driven gene networks in the key tissues involved in CRP/IL6, to detect top regulatory pathways and tissue-specific KDs in gene subnetworks that play a key role in regulating CRP and IL6 phenotypes. We applied an additional systems genomic approach to integrate independent GWAS data [27,28] for BC development with multi-omics datasets and explored the gene sets in enriched molecular subnetworks that overlap with those in CRP/IL6-relevant gene networks. Our multi-omics data analyses may reveal hidden mechanisms that are not apparent from individual top GWAS signals alone, by identifying the topmost significant molecular pathways and by detecting the full range of the functionalities of key genes in the subnetworks and their regulation from strong to subtle, which are reflected by the genetic perturbations of CRP/IL6. We further explored potential molecular mechanisms shared by CRP/IL6 and BC development. Our findings may thus provide system-level novel insights into CRP/IL6 from a molecular perspective and potential preventive and/or therapeutic strategies for associated diseases such as BC.

## 2. Materials and Methods

### 2.1. GWAS Data for CRP and IL6

Full details of the Women’s Health Initiative (WHI) database of Genotypes and Phenotypes (dbGaP) are given elsewhere [29,30]. We used the data of GWAS for CRP and IL6 [26] that we previously performed by using the WHI Harmonized and Imputed GWASs coordinated by the dbGaP in a joint imputation and harmonization effort across 6 GWASs within the WHI study [30]. Healthy postmenopausal women (age ≥ 50 years) were enrolled in the WHI study at >40 designated clinical centers in the U.S. from 1993 to 1998 and had been followed up through 29 August 2014, with a 16-year mean follow-up. All participants provided written informed consent. Our earlier GWAS included 10,798 women who reported their race or ethnicity as non-Hispanic white, with which we performed a GWAS meta-analysis for CRP and IL6 across the 6 GWASs for G × E interactions. Our study was approved by the institutional review boards of each participating clinical center of the WHI and by the University of California, Los Angeles.

### 2.2. Genotyping and CRP/IL6 Phenotypes

Genome-wide genotyping was performed at the Fred Hutchinson Cancer Research Center in Seattle, WA, with different platforms across the WHI GWASs, and further normalized to Genome Reference Consortium Human Build 37, imputed via 1000 genomes reference panels, and harmonized with pairwise concordance among all samples [31]. The minimum cutoff of allele frequency averaged 1.5% across the GWASs. A total of 21,784,812 autosomal single-nucleotide polymorphisms (SNPs) were analyzed in our GWAS by adjusting for age and 10 genetic principal components [26]. The tested phenotypes include fasting serum levels of CRP (in mg/L) and IL-6 (in pg/mL).

### 2.3. Mergeomics

To detect gene sets in molecular pathways and key regulators in gene networks that were perturbed by genetic variations associated with CRP/IL6, we used Mergeomics [32], a robust computational pipeline that integrates multi-omics datasets such as statistical summaries of phenotype associations and molecular networks. Mergeomics has been shown to outperform other gene set–enrichment methods [32]. For example, it can overcome heterogeneity between datasets from different studies, providing confirmatory biologic signals across data types and studies.

#### 2.3.1. Mapping SNPs to Genes

To link GWAS signals to SNPs in the pathways, we used two different mapping methods and generated two sets of SNP–gene maps: chromosomal distance– and whole blood eQTL–based mapping. First, standard chromosomal distance–based mapping to genes was used to generate a distance-based map within 50 kb of the gene region. Next, we used eQTL-based mapping, which contains expression single-nucleotide polymorphisms (eSNPs) associated with gene expression (i.e., eQTLs). The eSNPs within the eQTLs can capture the functional relationships between GWA SNPs and expressed genes in a tissue-specific manner. We used whole-blood eQTL–based mapping because it mainly reflects gene regulation in immune cells [19]. We selected *cis*-eSNPs (within 1 Mb of the gene region at a false discovery rate [FDR] < 0.05) to detect mechanistic clues in peripheral blood mononuclear cells where the gene expression intersected the CRP/IL6-eSNPs. In addition, we corrected linkage disequilibrium (LD) structure by including SNPs with strong associations with phenotypes in LD (R^2^ > 0.5).

#### 2.3.2. Marker-Set Enrichment Analysis (MSEA)

Our MSEA approach was based on canonical pathways that are largely derived from biochemical reactions, cellular signaling, and functional categories and that are relatively straightforward to annotate corresponding gene networks. We applied a knowledge-based pathway approach using 1827 canonical pathways from the Reactome, Biocarta, and the Kyoto Encyclopedia of Genes and Genomes (KEGG) databases [33,34]. We conducted the MSEA using the Mergeomics package, a well-established pathway method, to test for enrichment of genes for CRP/IL6 in the relevant pathway on the basis of modified chi-square statistics which adapt the summarized cutoff of the *p* value over a range of quantiles of marker selections [32,35]. A FDR < 0.05 was chosen to be statistically significant.

To capture core gene sets from overlapping pathways across the two phenotypes, we used the Meta-MSEA approach in Mergeomics to perform meta-analysis. With identified pathways within the Meta-MSEA framework, we further constructed independent supersets by combining overlapping pathways with gene overlap ratio *r* > 0.15 and FDR < 0.05 and finally created functionally categorized supersets (seven for distance mapping and eight for eQTL mapping).

#### 2.3.3. Tissue-Specific Gene Regulatory Networks and Weighted KD Analysis (wKDA)

Next, we applied the wKDA strategy in the Mergeomics pipeline to detect key regulator genes within the CRP/IL6 supersets, which are mapped to gene-regulatory subnetworks; these key genes thus potentially lead to the regulation of CRP/IL6 in molecular biologic cascades. For this analysis, we used (a) Bayesian gene-regulatory networks composed of human transcriptome datasets and known functional gene relationships from blood and adipose, liver, and muscle tissues and (b) protein–protein interaction networks (PPIs) [36,37]. We performed the wKDA [32,38,39] to identify KDs whose network neighbors are enriched for genes in the supersets on the basis of modified chi-square statistics [32,35] at FDR < 0.05. Thus, the topmost KDs are potential regulators of CRP/IL6-related genes and the phenotypes themselves.

#### 2.3.4. MSEA and wKDA for BC Development

Finally, we conducted an additional MSEA and wKDA within Mergeomics using independent GWAS data [27,28] for BC development. We investigated potential molecular pathways and KDs in subnetworks that are shared with those for CRP/IL6.

## 3. Results

### 3.1. Phenotype-Specific Pathways and Common Supersets Shared by CRP and IL6

First, we performed phenotype-specific MSEA for CRP and IL6 in distance-based and eQTL-based mapping. Among those significant pathways with a FDR < 0.05 for the enrichment of gene sets for CRP (Tables S1 and S2), 23 pathways were shared by distance-based (22% of 103 gene sets) and eQTL-based mapping (32% of 71 gene sets) (Figure S2). These included hematopoietic cell lineage; peptide hormone, amino sugar, and lipid metabolisms; calcium-dependent skeletal myogenesis; and cytokine signaling in the immune system. For IL6-specific pathways (Tables S3 and S4), 11 common pathways were found among the significantly enriched pathways (FDR < 0.05) that overlapped between distance-based (10% of 114 gene sets) and eQTL-based (20% of 55 gene sets) mapping (Figure S3). As was the case in the CRP-specific pathways, amino acid transport/metabolism was observed in the IL6-specific pathways. The IL6-unique pathways included signaling by receptor tyrosine kinase erb-b4 (ERBB4), *O*-glycosylation of mucins that have antimicrobial activity as a mucosal barrier, and transcription function such as metabolism of non-coding RNA. Involvement of CRP and IL6 in the immune system is not surprising considering that they function as inflammatory markers.

**Figure 1:**
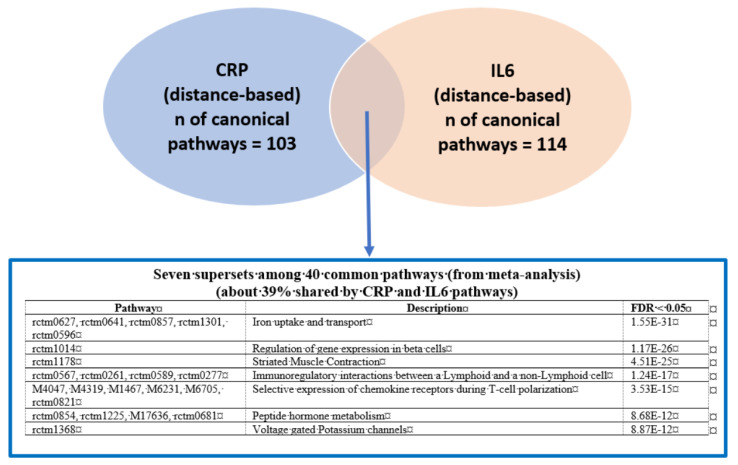
Comparison of significant pathways (false discovery rate [FDR] < 0.05) between C-reactive protein (CRP) and interleukin-6 (IL6) phenotypes (CRP/IL6, 50-kb distance–based mapping).

**Figure 2:**
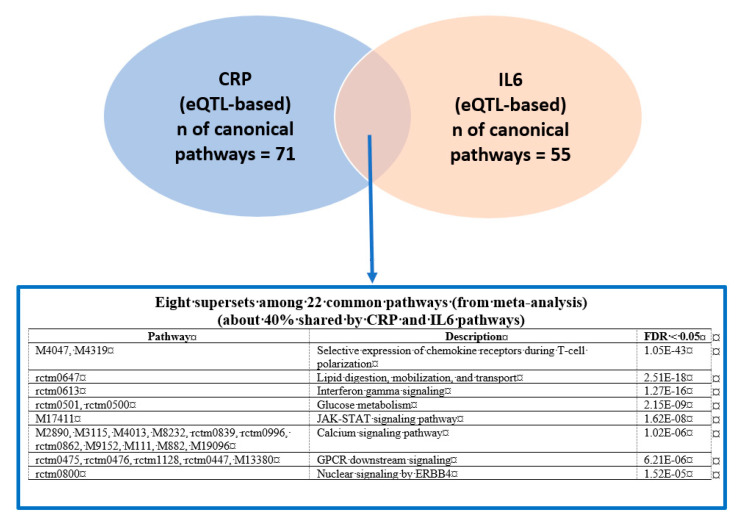
Comparison of significant pathways (false discovery rate [FDR] < 0.05) between C-reactive protein (CRP) and interleukin-6 (IL6) phenotypes (CRP/IL6, expression quantitative trait loci [eQTL]–based mapping; GPCR, G protein–coupled receptor; JAK-STAT, Janus kinase-signal transducer and activator of transcription.).

We next conducted a meta-MSEA across CRP and IL6 in either distance-based or eQTL-based mapping to detect core gene sets that were enriched in the shared pathways. Because the knowledge-driven molecular pathways identified through meta-analysis have redundant gene sets on similar functions, we combined the overlapped pathways into functionally categorized independent supersets. In particular, distance mapping–based meta-MSEA (Figure 1) detected seven supersets (FDR < 0.05) among 40 common individual pathways that were shared about 40% by CRP and IL6, including well-known CRP/IL6 pathways (e.g., immunoregulatory interactions between lymphoid and non-lymphoid cells and selective expression of chemokine receptors during T-cell polarization) as well as lesser-known pathways, including gene expression in pancreatic beta cells, striated muscle contraction, and peptide hormone and iron/potassium channels metabolism. In the eQTL mapping–based meta-MSEA (Figure 2), we found eight supersets (FDR < 0.05) that functionally merged 22 common individual pathways (40%) shared by CRP and IL6; of those, one (chemokine receptors during T-cell polarization) overlapped with the distance mapping–based supersets. The eQTL mapping–specific supersets included well-known immune pathways, such as interferon γ (IFNγ) and Janus kinase-signal transducer and activator of transcription (JAK-STAT) signaling, as well as general cellular pathways, such as calcium/G protein–coupled receptor (GPCR) signaling. Of note, the eQTL-based shared supersets included lipid and glucose metabolisms and nuclear signaling by ERBB4 (also known as human epidermal growth factor receptor 4 [HER4]), which play critical roles in carcinogenic progression of specific cancers, including BC.

We further conducted a meta-MSEA of both distance- and eQTL based–mapping types across CRP and IL6 (Figure S4), detecting 22 common pathways, most of which overlapped with those supersets identified from the meta-analysis in each mapping. In detail, they included cellular-based pathways (e.g., transforming growth factor-β); chemokine receptors and their signaling; immune responses (e.g., IL12/STAT4 signaling in T-helper 1 and IL10 signaling); ERBB4 signaling (e.g., *NOTCH1*); JAK-STAT signaling; and the downstream activation of ERBB4, JAK-STAT, and GPCR signaling (e.g., mitogen-activated protein kinase [MAPK]). These indicate that each mapping type contributes to revealing immune–response pathways in the meta-analysis across the two phenotypes, whereas eQTL-mapping provides more informative downstream signaling (e.g., ERBB4, JAK-STAT, GPCR), suggesting that functional eSNPs associated with gene expression in whole blood better captured cellular-level mechanisms that regulate CRP/IL6.

#### Putative KD Genes for the CRP/IL6–Associated Supersets

**Figure 3:**
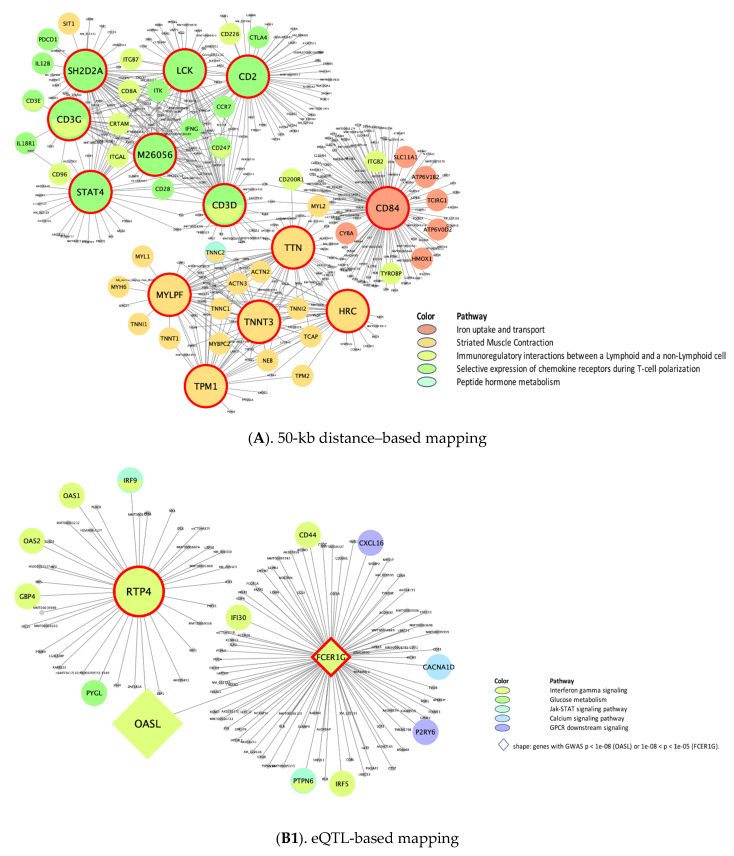

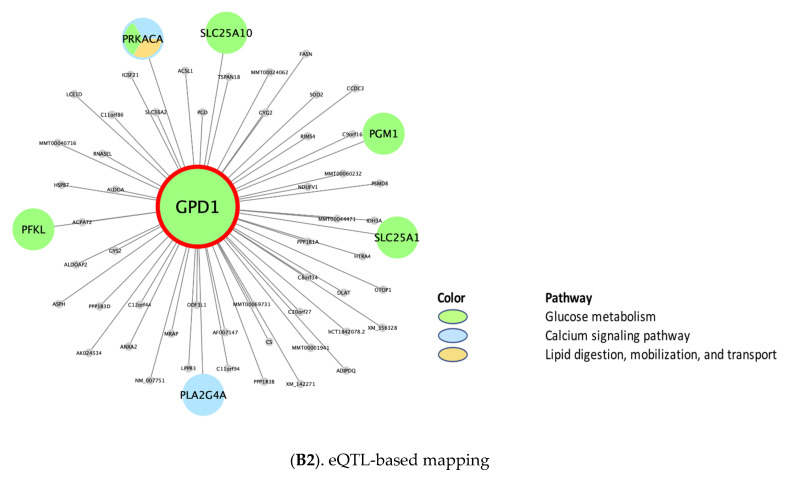
Adiposity-specific gene-regulatory networks of top KDs in meta-analysis of CRP and IL6. (CRP, C-reactive protein; eQTL, expression quantitative trait loci; IL6, interleukin-6; KD, key drivers. The bigger nodes with red outlines are the top KDs in the enriched pathway obtained from weighted KD analysis. The subnetworks of the KDs are indicated by different colors according to differences in their canonical functions.).

**Figure 4:**
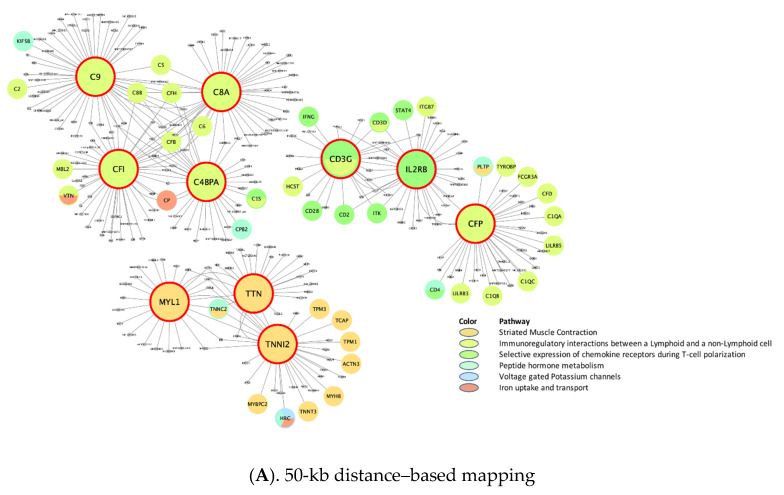

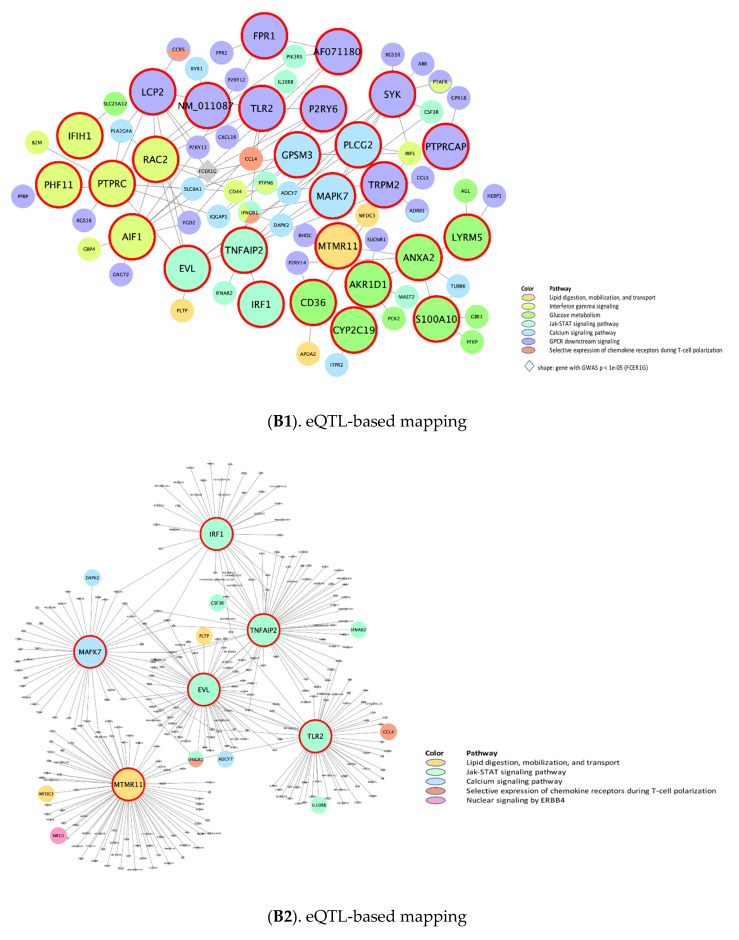
Liver-specific gene-regulatory networks of top KDs in meta-analysis of CRP and IL6. (CRP, C-reactive protein; eQTL, expression quantitative trait loci; IL6, interleukin-6; KD, key drivers. The bigger nodes with red outlines are the top KDs in the enriched pathway obtained from weighted KD analysis. The subnetworks of the KDs are indicated by different colors according to differences in their canonical functions.).

**Figure 5:**
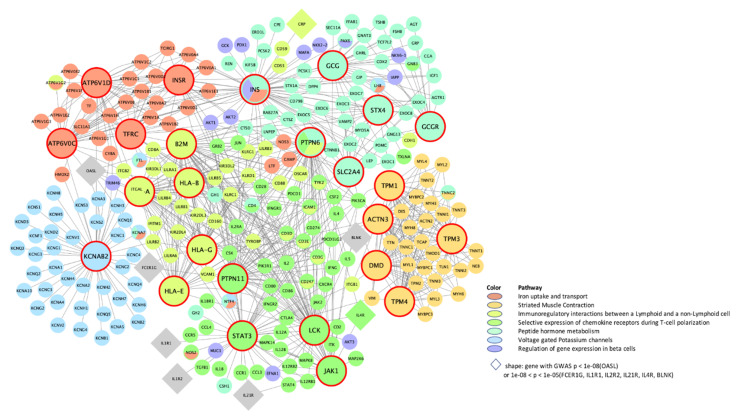
PPI-specific gene-regulatory networks of top KDs in meta-analysis of CRP and IL6 on 50-kb distance–based mapping. (CRP, C-reactive protein; IL6, interleukin-6; KD, key drivers; PPI, protein-to-protein interaction network. The bigger nodes with red outlines are the top KDs in the enriched pathway obtained from weighted KD analysis. The subnetworks of the KDs are indicated by different colors according to differences in their canonical functions.).

**Figure 6:**
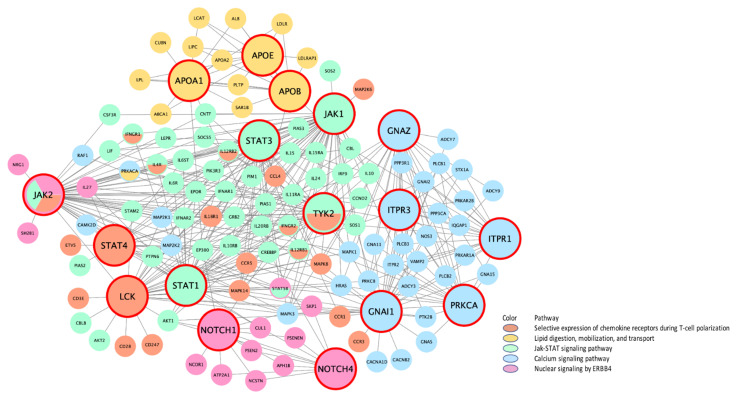
PPI-specific gene-regulatory networks of top KDs in meta-analysis of CRP and IL6 on eQTL-based mapping. (CRP, C-reactive protein; eQTL, expression quantitative trait loci; FDR, false discovery rate; IL6, interleukin-6; KD, key drivers; PPI, protein-to-protein interaction. The bigger nodes with red outlines are the top KDs in the enriched pathway obtained from weighted KD analysis. The subnetworks of the KDs are indicated by different colors according to differences in their canonical functions.).

With those supersets shared across CRP and IL6 in each mapping style, we subsequently performed wKDA to detect the important hub genes (i.e., KDs) that regulate neighbor genes in the G × G interaction subnetworks associated with CRP/IL6. We obtained tissue-specific KDs from PPIs as well as blood and adipose, liver, and muscle tissues, each of which reflects different molecular mechanisms regulating CRP/IL6. The tissue-specific topmost KDs for the supersets shared by CRP/IL6 (Table 1 and Table 2 for distance- and eQTL-based mapping, respectively) and their representative subnetworks (Figure 3, Figure 4, Figure 5 and Figure 6) are shown. In particular, adipose tissue–specific distance-based KDs (Table 1 and Figure 3A) in the immune regulations/chemokine receptor subnetworks include *CD3G*, *CD3D*, *CD2*, *LCK*, *SH2D2A*, and *STAT4*; and in the iron subnetwork, one primary KD, *CD84*, was detected. Those networks were interrelated mainly by *CD3D*. In adipose tissue–specific eQTL-based KDs (Table 2 and Figure 3(B1,B2)), two different subnetworks were involved: (a) *RTP4* and *FCER1G* in IFNγ signaling and (b) *GPD1* in glucose metabolism. Of note, the *RTP4* in the IFNγ subnetwork was connected to *FCER1G* by 1 gene (*OASL*); both *FCER1G* and *OASL* reached the genome-wide significance level.

In addition, liver tissue-specific distance-based KDs (Figure 4A) in the immune regulation/chemokine receptor subnetworks included *C9*, *CFI*, *C8A*, *C4BPA*, *CFP*, and *IL2RB*. It is interesting that *CD3G* is a KD shared by adipose and liver tissues in the immune regulatory networks. Also, similar to the wKDAs of adipose tissue-specific eQTL mapping, the liver tissue-specific eQTL-based wKDAs (Figure 4(B1)) yielded IFNγ and glucose metabolism subnetworks, but with different sets of KDs (*PTPRC*, *RAC2*, *PHF11*, and *IFIH1* for IFNγ; *ANXA2*, *AKR1D1*, *LYRM5*, *CYP2C19*, and *S100A10* for glucose metabolism). Further, those KDs were involved in the GPCR subnetwork. When the JAK-STAT subnetwork was enlarged (Figure 4(B2)), several KDs (*TNFAIP2*, *TLR2*, *IRF1*, and *EVL*) were found to be interrelated with calcium (KD: *MAPK7*) and lipid (KD: *MTMR11*) subnetworks.

Further, our PPI-specific wKDA in distance mapping (Figure 5) detected different sets of KDs than the adipose/liver tissue-specific distance-based wKDAs in the immune/chemokine receptor-regulatory subnetworks (*HLA–A/B/E/G* in immune regulations; *PTPN11/JAK1* in chemokine receptor expression). However, *LCK* and *STAT3* in the chemokine-receptor subnetwork overlapped with those in the corresponding adipose tissue-specific subnetwork. Those immune-regulation networks were interrelated with iron (KD: *ATP6V1D*, *ATP6V0C*, and *INSR*) and potassium channels (KD: *KCNAB2*) and had several GWA genes in their peripheral nodes. In eQTL-based PPI-specific wKDA (Figure 6), several KDs (*JAK1*, *STAT1/3/4*, and *LCK*) in the JAK-STAT signaling overlap with those in the chemokine-receptor subnetwork of both eQTL- and distance-based PPI wKDAs, implying their congruent roles in immune responses. Of note, *TYK2*, within the eQTL mapping, shares the two subnetworks of JAK-STAT and chemokine receptor regulations, and *JAK2* shares the three subnetworks, including ERBB4 signaling as well as the two aforementioned immune subnetworks.

We further performed a MSEA and subsequent wKDAs for BC development using an independent GWA dataset and detected pathways and KDs in subnetworks that were shared by gene supersets of CRP/IL6 (Table S5). Although ERBB4 signaling overlapped BC and CRP/IL6, no significant KD was detected in the BC subnetwork. In addition to glucose metabolism with *PYGB*, a KD gene overlapping BC and CRP/IL6, the BC pathways included immune regulatory interactions/chemokine receptor mechanisms/JAK-STAT signaling with their KDs (*CD3D*, *CD3G*, *IL2*, *IL4, JAK1/2/3*, *STAT6*, and *TYK2*), all of which overlapped key regulatory genes in the CRP/IL6-assocaited gene networks.

## 4. Discussion

Accumulating evidence from population-based genomic studies [20,40,41] supports that multiple genes together in biological pathways and gene-regulatory networks, compared with the individual genes, coordinate better in revealing the underlying mechanism of quantitative phenotypes and disease risks. For this reason, we integrated the GWAS data from standard GWAS analyses with multi-omics data, including eQTLs, knowledge-based canonical pathways, and tissue-based gene networks and detected diverse sets of genes within the biologic pathways across CRP and IL6 phenotypes. Also, among the hundreds of genes involved in the particular pathways, we identified important gene regulators of the topmost significant pathways to prioritize genes and uncover novel regulatory mechanisms of CRP/IL6 that may not have been detected without such a systems-biology study.

In detail, we identified several shared pathways across the two phenotypes in distance- and eQTL based-mapping, both separately and together. In particular, immune regulatory interactions between non-lymphoid and lymphoid cells clearly reflect the IL6/CRP immune responses. For example, activated macrophages, one of the non-lymphoid immune-regulation cells, produce proinflammatory cytokines, including IL6 [42]. IL6 is the main stimulator of other inflammatory proteins/cytokines such as CRP, largely by promoting their production in hepatocytes [43]. Next, by combining with its soluble receptor (sIL-6Rα), IL6 elicits the development of cellular-specific immune responses, including end-stage B-cell differentiation and T-cell activation [44]. Thus, IL6 is important in the transition between non-lymphoid and lymphoid immune regulation. In addition, the IFNγ signaling detected in our study mediates inflammation and cell-mediated immune responses. For example, IFNγ activates macrophages, which in turn induce cytokines (e.g., CRP/IL6) to facilitate accumulation of immune cells at inflammation sites [45]. IFNγ signaling is also involved in cell differentiation and apoptosis and in anti- and pro-tumorigenesis. In detail, low expression of major histocompatibility complex (MHC) antigen leads tumor cells to evade the host immune response against tumor cells, and treatment with IFNγ activates the MHC antigens, resulting in tumor regression [46]. IFNγ treatment also upregulates chemokines such as CXCL9 and CXCL10 [47,48], which are important in recruiting T cells into tumors of various origin, including breast, colorectum, ovary, and lung [49,50]. By contrast, IFNγ has a pro-tumorigenic effect by interacting with a number of chemokines, activating JAK-STAT signaling, which leads to increased programmed death-ligand 1 (PD-L1) surface display [51,52,53]. A large amount of PD-L1 in cancer sites allows cancer cells to evade T cells’ anti-tumor activity.

As noted above, chemokines and their receptors and JAK-STAT signaling, both of which were associated with CRP/IL6 in our study, are involved in the inflamed immune cell–enriched tumor microenvironment [50,54]. Particularly, CXCR3 chemokine receptor, along with ligands CXCL9 and CXCL10, is a major regulator of T cells infiltration to target tumor cells [55]. One of our KDs detected in the chemokine receptor process is CXCR3, which is a promising target for regulating immune-related tumorigenesis. Also, JAK-STAT signaling reflects the protein–protein interactions in a cell that are largely involved in immunity, cell division, apoptosis, and tumor formation [56]. Specifically, JAK-STAT signaling, triggered by the IL6/sIL-6Rα complex, activates extracellular signal-regulated protein kinase 1 and 2 and phosphoinositide 3-kinase downstream pathways that have been implicated in tumor cell growth [54,57,58,59]. Further, a JAK2 inhibitor, blocking IL6/JAK-STAT signaling, represses the secretion of CRP [60], confirming our finding that this pathway is well linked to IL6/CRP phenotypes.

Moreover, we detected ERBB4 signaling as one of the topmost significant pathways shared by CRP/IL6 and BC development; this finding is supported by previous studies reporting that (a) human pro-inflammatory macrophages stimulating CRP/IL6 production induces ERBB4 expression and (b) CRP/IL6 correlates with breast tissue aromatase levels in women diagnosed with BC and those with a high risk of BC [61]. Finally, ERBB4 has been inhibited as a therapeutic target for BC by pan-ERBB tyrosine kinase inhibitors such as lapatinib [62]. The KDs NOTCH1 and NOTCH4 that we detected in the ERBB signaling subnetwork can be further studied as novel targets for modulating the CRP/IL6 axis and the risk of BC.

With those topmost pathways associated with CRP/IL6, we further examined regulatory connections between genes and pathways in G × G interaction networks. Identifying KD genes in a tissue-specific manner in the gene networks can uncover key regulatory components in an effort to identify tissue-specific drug targets and biomarkers for CRP/IL6 and associated diseases such as BC. We detected many known regulators in the pathways of immune regulation/chemokine receptor expression: *CD3D*, *CD3G*, *CD2*, *LCK*, and *SH2D2A* in adipose tissue and *IL2RB* in liver tissue, all of which are associated with the T-cell receptor/CD3 complex and thus involved in T-cell development and signal transduction [63,64,65,66,67,68]; *C8A*, *C9*, *CFI*, and *CFP* in liver tissue, which encode the components of the complement pathways in the immune system [69,70,71,72]; and *HLA-A/B/E/G* in PPI-specific subnetworks, which belong to the MHC Class I, leading cytotoxic T cells to recognize peptides from the endoplasmic reticulum membranes [73,74,75,76]. Among all of these, *CD3D* and *CD3G*, forming CD3-delta and gamma complex, respectively, were found in our study as KDs shared by BC development. Whereas several studies have reported the role of CD3 cells in promoting immunosuppressive capacity in hepatocellular [77], urothelial [78], and non–small cell lung [79] carcinomas, studies for BC risk are lacking, warranting further studies of those regulatory genes as promising drug targets in immune-associated BC carcinogenesis.

Also, we detected well-established genes (e.g., RTP4, FCER1G, and IRF1) [80,81,82,83] in IFNγ signaling. In particular, IRF1 was detected in both IFNγ and JAK-STAT signaling; this finding is supported by the previous report that IRF1 encodes IFN regulatory factor 1, which is involved in IL12 signaling mediated by STAT [82]. In addition, we found key regulatory genes in JAK-STAT signaling, many of which overlapped immune regulatory chemokine receptor–associated genes, indicating their great involvement in the immune system. JAK1/2/3 are specifically targeted by FDA-approved drugs such as tofacitinib and ruxolitinib for treatment of autoimmune diseases [84,85,86]. Also, STAT3 leads to the transcription of IL6-responsive genes, resulting in leukocyte infiltration and inflammation [87,88]; thus, it has been served as an effective drug target for malignant and inflammatory diseases [54]. In our study, we detected STAT6 in addition to JAK1/2/3 as KDs shared by BC development. Previous studies have revealed that STAT6 exerts its effect via IL4-mediated biologic responses [89], is involved in impairing metastasis of BC cells to the lungs [90], and is associated with a better prognosis for BC patients [91]. This suggests a critical role of STAT6 in immune-mediated cancer progression, and it can thus be considered a promising drug target or biomarker for BC prevention and/or treatment.

The GWAS database we used in this study may not capture the full array of unknown biology related to CRP/IL6/BC risk. Also, we omitted directional analyses and did not detect epistatic interactions among the regulatory genes. Further, our data focused on individuals of European ancestry, so the generalizability of our findings to other populations is limited. Nonetheless, our study has detected well-known pathways and KDs related to the phenotypes, which have been targeted by FDA-approved drugs, indicating that our systematic multi-omics data approach is robust and productive. In addition, consistent with previous findings [35,92], most of the KDs we detected were not the top GWAS hits, supporting evolutionary constraints [93,94]. However, those KDs that have central properties in the gene networks exert strong effects on phenotype regulation and associated-disease risk, thus they may be better candidates for drug targets and biomarkers.

## 5. Conclusions

Overall, we detected both shared and unique biologic pathways across CRP/IL6 and BC development. The gene-regulatory networks enriched by CRP/IL6 pathways revealed in a tissue-specific manner a number of key driver genes, of which both well-established (e.g., JAK1/2/3 and STAT3) and novel (e.g., CXCR3, *CD3D, CD3G,* and STAT6) drug targets were recognized for their shared mechanisms in regulating CRP/IL6 and BC risk. Our study, if validated in an independent large genomic study, may contribute to the better revelation of novel genetic targets for CRP/IL6 regulation, which would enable preventive and therapeutic strategies for the associated disorders, such as BC.

## Figures and Tables

**Table 1:** MSEA Meta-analysis of CRP and IL6 pathways (distance-based mapping) and corresponding tissue specific–network key drivers.

			Top 10 Key Drivers £				
Module €	Description	Module Size	Adipose	Blood	Liver	Muscle	PPI
rctm0567 ..	Immunoregulatory interactions between lymphoid and non-lymphoid cells	120 **, 70 ¶, 126 ¥, N/A, 126 §	*CD3G* *, CD2, LCK, STAT4	*CD8B* *	*C4BPA* *, C8A *, C9 *, CFI *, CFP, CSF1R, CFH *, C8B *, C5 *, CFB *	N/A	*HLA-A, B2M* *, HLA-E, HLA-G, HLA-B, HLA-C, CD28, CD80, C4A, HLA-F
M4047 ..	Selective expression of chemokine receptors during T-cell polarization	76 **, N/A, 80 ¥, N/A, 83 §	*CD3D* *, M26056, SH2D2A, LCK *, CD2 *, CD3G *, TNFRSF18, CD5, ITGAL, CXCR3	N/A	*CD3G* *, IL2RB	N/A	*STAT3, LCK* *, PTPN11 *, JAK1, PTPN6 *, PIK3CA *, IL4 *, JAK2 *, IL2 *, SHC1
rctm0627 ..	Iron uptake and transport	62 **, N/A, N/A, N/A, 62 §	*DPEP2, PLD3, CD84, ATF3*	N/A	N/A	N/A	*TFRC* *, INS *, ATP6V1D *, ATP6V0C *, INSR *
rctm0854 ..	Peptide hormone metabolism	N/A, N/A, N/A, N/A, 121 §	N/A	N/A	N/A	N/A	*INS* *, STX4, SLC2A4, GCG *, GCGR, AVPR2, RHO, VAMP2 *, NPS, SNAP23
rctm1368	Voltage-gated potassium channels	N/A, N/A, N/A, N/A, 54 §	N/A	N/A	N/A	N/A	*KCNAB2* *
rctm1178	Striated muscle contraction	60 **, N/A, 53 ¥, 50 †, 53 §	*MYLPF, TTN* *, HRC, TPM1 *, TNNT3 *, TNNI2 *, TNNC2 *, MYPN, KBTBD10, TCAP *	N/A	*MYL1* *, TTN *, TNNI2 *	*TPM3* *, TNNI1 *, MYL2 *, MYL3 *, TNNT1 *, TNNC1 *, MYH3 *, TPM1 *, CYFIP2, ATP2A2, MYH7	*DMD* *, TPM1 *, ACTN3 *, TPM4 *, TPM2 *, ACTN2 *, TPM3 *, ACTA1, ITGA1, MYL9

CRP, C-reactive protein; IL6, interleukin 6; MSEA, marker-set enrichment analysis; N/A, not available; PPI, protein-to-protein interaction network. € Modules marked with two periods (..) are those that are merged. £ Key drivers are presented in ascending order of false discovery rate. ** Number of genes in adipose-specific network pathways. ¶ Number of genes in blood-specific network pathways. ¥ Number of genes in liver-specific network pathways. † Number of genes in muscle-specific network pathways. § Number of genes in PPI-based network pathways. * Member gene of the particular pathway in tissue-specific gene-regulatory network analysis.

**Table 2:** MSEA Meta-analysis of CRP and IL6 pathways (whole blood eQTL mapping) and corresponding tissue specific–network key drivers.

			Top 10 Key Drivers £				
Module €	Description	Module Size	Adipose	Blood	Liver	Muscle	PPI
M4047 ..	Selective expression of chemokine receptors during T-cell polarization	N/A, N/A, N/A, N/A, 20 §	N/A	N/A	N/A	N/A	*STAT3, JAK1, STAT4, STAT1, LCK, IL4, JUN, IL1B, STAT6, JAK2* *
rctm0613	Interferon gamma signaling	26 **, N/A, 26 ¥, 22 †, 28 §	*RTP4, FCER1G*	N/A	*PTPRC, RAC2, PHF11, AIF1, IFIH1, IFI44, FCGR1A, THEMIS2, CSF1R, ISG15*	*RTP4*	*SP100, HLA-A, IRF1, HLA-DQA1, IRF2, B2M* *, IRF9 *, FCGR1A, IRF7, HLA-F
M17411	JAK-STAT signaling pathway	N/A, N/A, 48 ¥, N/A, 49 §	N/A	N/A	*TNFAIP2, TLR2, IRF1, LCP2, EVL, ZFP90, RAC2*	N/A	*JAK1* *, JAK2 *, STAT3 *, STAT1, TYK2 *, JAK3, STAT5A, STAT6, IL4, PTPN11
rctm0800	Nuclear signaling by ERBB4	N/A, N/A, N/A, N/A, 23 §	N/A	N/A	N/A	N/A	*NOTCH1, NOTCH4*
rctm0647	Lipid digestion, mobilization, and transport	N/A, N/A, 25 ¥, N/A, 25 §	N/A	N/A	*MTMR11, CD36*	N/A	*APOB, APOA1, APOE*
rctm0501 ..	Glucose metabolism	31 **, N/A, 30 ¥, N/A, 31 §	*GPD1*	N/A	*ANXA2, AKR1D1, LYRM5, CYP2C19, S100A10, EHHADH, SLC47A1, ERMP1, SLC10A1, CD36*	N/A	*PGM1* *, ENPP1, GSK3B, PYGB *, PPP2R5C, PPP1CA, PPP2CB *
M2890 ..	Calcium signaling pathway	N/A, N/A, 67 ¥, N/A, 76§	N/A	N/A	*MAPK7, PLCG2, SYK, P2RY6, GPSM3, ARHGDIB*	N/A	*PRKCA* *, ITPR1 *, GNAZ *, ITPR3 *, GNAI1, ITPR2 *, HRAS *, GNAO1, GNB5, MAPK3 *
rctm0475 ..	GPCR downstream signaling	N/A, N/A, 156 ¥, N/A, 183 §	N/A	N/A	*PTPRCAP, FPR1* *, NM_011087, TRPM2, AF071180, AK016231, X51547, U96688, X15592, PIK3R5	N/A	*S1PR4, C5AR1, CCL5* *, CXCL10, CX3CR1, GPR18 *, CCR7, GPR183, CCL21, P2RY14 *

CRP, C-reactive protein; eQTL, expression quantitative trait loci; GPCR, G protein–coupled receptor; IL6, interleukin 6; JAK-STAT, Janus kinase-signal transducer and activator of transcription; MSEA, marker-set enrichment analysis; N/A, not available; PPI, protein-to-protein interaction network. € Modules marked with two periods (..) are those that are merged. £ Key drivers are presented in ascending order of false discovery rate. ** Number of genes in adipose-specific network pathways. ¥ Number of genes in liver-specific network pathways. † Number of genes in muscle-specific network pathways. § Number of genes in PPI-based network pathways. * Member gene of the particular pathway in tissue-specific gene-regulatory network analysis.

## Data Availability

The data that support the findings of this study are available in accordance with policies developed by the NHLBI and WHI in order to protect sensitive participant information and approved by the Fred Hutchinson Cancer Research Center, which currently serves as the IRB of record for the WHI. Data requests may be made by emailing helpdesk@WHI.org, accessed on 1 January 2021.

## Supplementary Materials

The following are available online at https://www.mdpi.com/article/10.3390/biom11091379/s1, Figure S1: Schematic diagram of the study. (CRP, C-reactive protein; eQTL, expression quantitative trait loci; GWAS, genome-wide association study; IL6, interleukin-6; MSEA, marker-set enrichment analysis; PPI, protein-to-protein interaction; SNP, single nucleotide polymorphism). Figure S2: Comparison of significant pathways (false discovery rate [FDR] < 0.05) for C-reactive protein (CRP) phenotype between 50-kb distance–based and expression quantitative trait loci [eQTL]–based mapping. Figure S3: Comparison of significant pathways (false discovery rate [FDR] < 0.05) for interleukin 6 (IL6) phenotype between 50-kb distance–based and expression quantitative trait loci [eQTL]–based mapping. Figure S4: Comparison of significant pathways (false discovery rate [FDR] < 0.05) between C-reactive protein (CRP) and interleukin-6 (IL6) phenotypes across distance–based and expression quantitative trait loci [eQTL]–based mapping. Note: The yellow-highlighted pathways overlap with those from the meta-analysis of CRP and IL-6 in either mapping or their downstream cascades. Table S1: MSEA analysis of CRP pathways (CRP, 50-kb distance–based mapping; pathways arranged by ascending FDR). Table S2: MSEA analysis of CRP pathways (CRP, eQTL-based mapping; pathways arranged by ascending FDR). Table S3: MSEA analysis of IL6 pathways (IL6, 50-kb distance–based mapping; pathways arranged by ascending FDR). Table S4: MSEA analysis of IL6 pathways (IL6, eQTL-based mapping to genes; pathways arranged by ascending FDR). Table S5: Tissue-specific shared pathways and KD genes across BC development and CRP/IL6.
