# Supplementary material for: Molecular Biology Networks and Key Gene Regulators for Inflammatory Biomarkers Shared by Breast Cancer Development: Multi-Omics Systems Analysis
Source: Biomolecules. 2021 Sep 18;11(9):1379. doi: 10.3390/biom11091379 (PMC8469138; doi:10.3390/biom11091379)
Supplement: Supplementary file 1 [file biomolecules-11-01379-s001.zip › biomolecules-1339199-supplementary.pdf]

Figure S1. Schematic diagram of the study. (CRP, C-reactive protein; eQTL, expression quantitative trait loci; GWAS, genome-wide association study; IL6, interleukin-6; MSEA, marker-set enrichment analysis; PPI, protein-to-protein interaction; SNP, single nucleotide polymorphism)

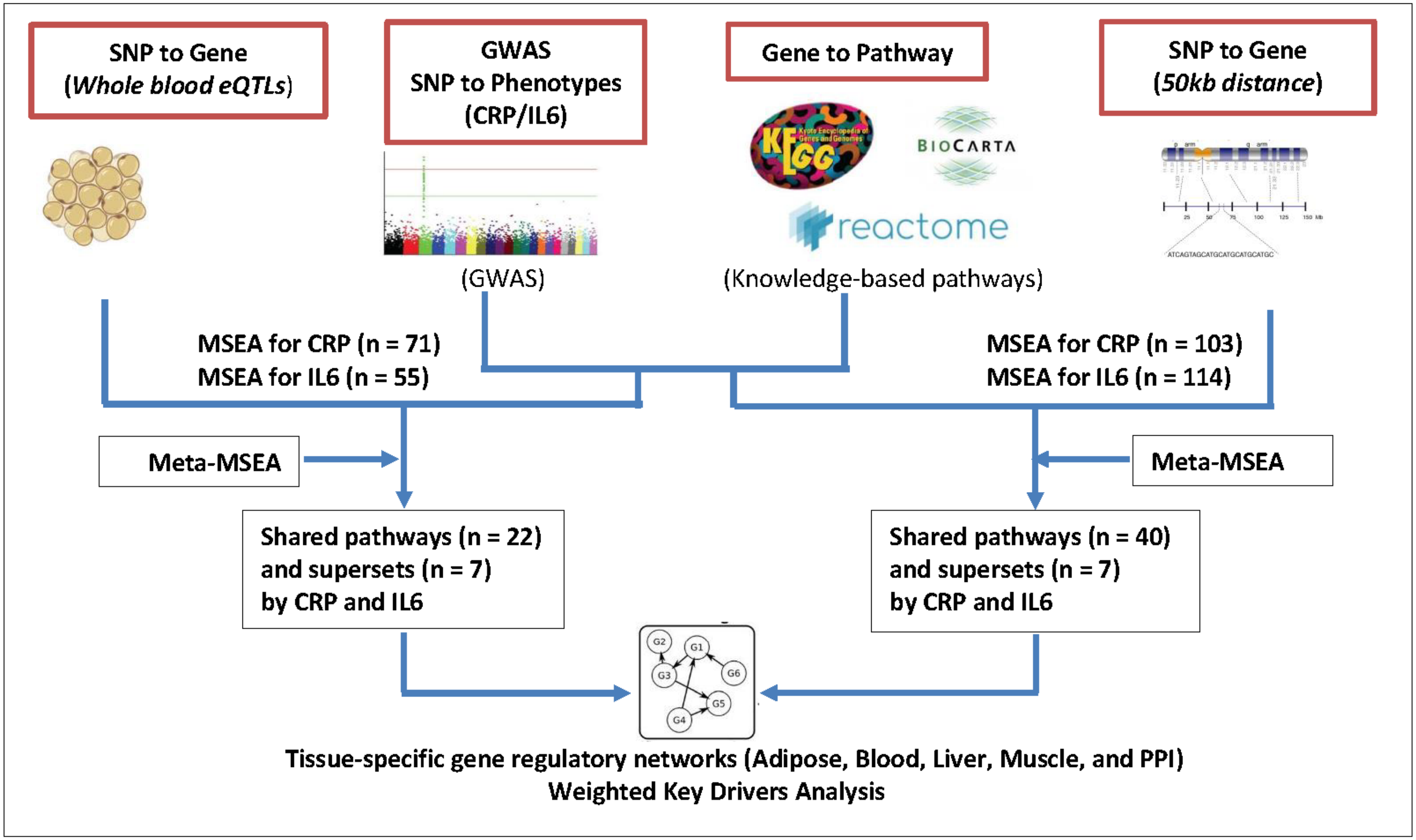

Figure S2. Comparison of significant pathways (false discovery rate [FDR] < 0.05) for C-reactive protein (CRP) phenotype between 50-kb distance–based and expression quantitative trait loci [eQTL]–based mapping.

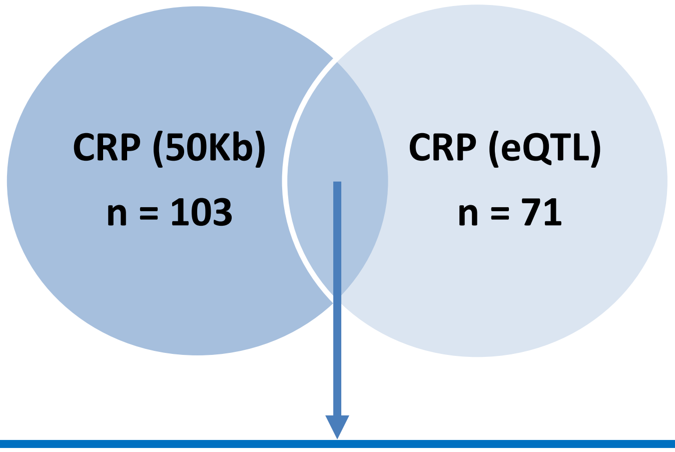

Twenty-three common pathways
(about 22% of CRP-50 kb and 32% of CRP-eQTL)

Pathway	Description	Pathway	Description
M12039	One carbon pool by folate	rctm0709	Mitochondrial protein import
rctm0495	Generic transcription pathway	rctm0678	Membrane trafficking
rctm0798	Nuclear receptor transcription pathway	rctm0854, rctm0594	Peptide hormone metabolism
M5109, M14314, rctm0690, M1724, rctm0929, M17726, rctm0961	Pyrimidine metabolism	M17400, rctm1113	ALK in cardiac myocytes
AD_Positive	Positive control for AD	M4383	Actions of nitric oxide in the heart
rctm0602, rctm0871, rctm0601	Integrin cell surface interactions	M1547, M5290	Control of skeletal myogenesis by HDAC and calcium/calmodulin-dependent kinase (CaMK)
M6856	Hematopoietic cell lineage	M8104	Amino sugar and nucleotide sugar metabolism
rctm0457	G0 and early G1	rctm0933, M3985	Pyruvate metabolism and citric acid (TCA) cycle
rctm0517	Golgi cisternae pericentriolar stack reorganization	rctm0647	Lipid digestion, mobilization, and transport
rctm0238, rctm1256, rctm0463, rctm0044, M16853, rctm0307, M13515, rctm0387, rctm0639, rctm1253	Chromosome maintenance	rctm0289, rctm0611, rctm0613, rctm0612	Cytokine signaling in immune system
rctm1346, rctm0055, rctm0053	Unfolded protein response	M9052	Phosphatidylinositol signaling system
M6981	Protein export		

Figure S3. Comparison of significant pathways (false discovery rate [FDR] < 0.05) for interleukin 6 (IL6) phenotype between 50-kb distance–based and expression quantitative trait loci [eQTL]–based mapping.

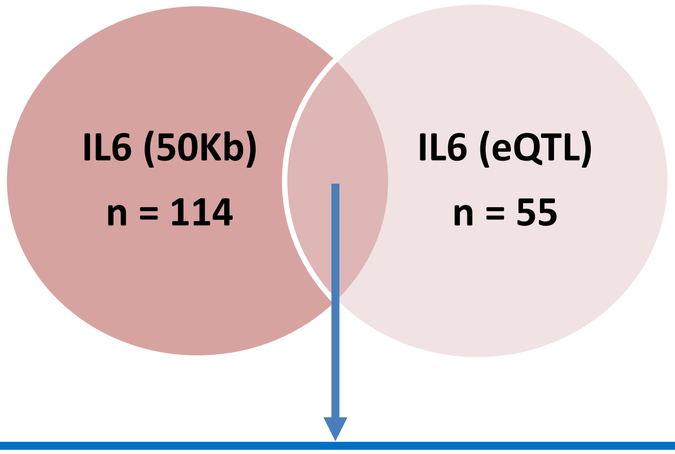

Eleven common pathways
(about 10% of IL6-50 kb and 20% of IL6-eQTL)

Pathway	Description	Pathway	Description
M2044, rctm1398, rctm1026, rctm0701	Spliceosome	rctm0805, M6929, rctm0629, rctm1258	*O*-linked glycosylation of mucins
M16743, M7151, M12524	Tyrosine metabolism	M7528	Pantothenate and CoA biosynthesis
M4361	Proximal tubule bicarbonate reclamation	M11266	Lysosome
M1519	Endocytosis	rctm0709	Mitochondrial protein import
rctm1327, rctm0106, rctm0108	Transport of inorganic cations/anions and amino acids/oligopeptides	rctm0689, rctm1415, rctm0721, rctm0517	Metabolism of non-coding RNA
rctm1117, rctm1130, rctm0800, rctm0519	Signaling by ERBB4		

Figure S4. Comparison of significant pathways (false discovery rate [FDR] < 0.05) between C-reactive protein (CRP) and interleukin-6 (IL6) phenotypes across distance–based and expression quantitative trait loci [eQTL]–based mapping. Note: The yellow-highlighted pathways overlap with those from the meta-analysis of CRP and IL-6 in either mapping or their downstream cascades.

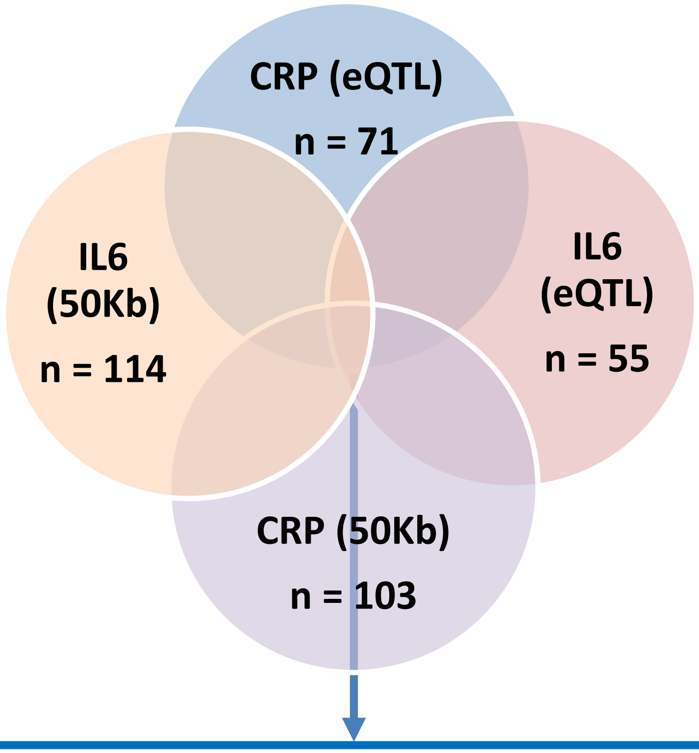

Twenty-one common pathways (from meta-analysis) shared by CRP and IL6 across distance–based and eQTL–based mapping

Pathway	FDR < 0.05
Defects in vitamin and cofactor metabolism	3.51E-86
Downregulation of TGF-beta receptor signaling	4.29E-71
Initial triggering of complement	2.06E-69
Regulation of beta-cell development	9.49E-65
TGF-beta receptor signaling activates SMADs	9.04E-64
Mitochondrial protein import	2.11E-61
Integrin signaling	1.72E-60
Platelet aggregation (plug formation)	1.08E-56
Notch-HLH transcription pathway	1.81E-49
Chemokine receptors bind chemokines	7.38E-47
Complement cascade	1.14E-43
Selective expression of chemokine receptors during T-cell polarization	2.89E-42
Chemokine signaling pathway	2.24E-40
Signaling by NOTCH1	2.61E-40
IL12- and STAT4-dependent signaling pathway in Th1 development	1.24E-37
Interleukin-10 signaling	5.02E-35
SUMOylation of DNA damage response and repair proteins	9.79E-28
Transcriptional regulation by RUNX1	2.94E-21
Golgi cisternae pericentriolar stack reorganization	4.68E-21
JAK-STAT signaling pathway	1.94E-20
MAP2K and MAPK activation	1.77E-16

Table S1. MSEA analysis of CRP pathways (CRP, 50-kb distance–based mapping; pathways arranged by ascending FDR)

**Module* (n=103)**	**Description**	**FDR < 0.05**	**n of genes**	**n of SNPs**
M17844	Other glycan degradation	4.67E-24	16	2,184
rctm1363	Vitamin B5 (pantothenate) metabolism	5.22E-21	11	1,437
rctm0289..	Cytokine signaling in immune system	8.81E-21	105	12,989
rctm0238..	Chromosome maintenance	1.03E-20	94	12,810
rctm0411	Factors involved in megakaryocyte development and platelet production	1.04E-20	114	18,138
rctm0104	Amine-derived hormones	7.10E-20	15	2,332
rctm0255	Cobalamin (Cbl, vitamin B12) transport and metabolism	1.38E-19	19	3,178
rctm0567..	Immunoregulatory interactions between lymphoid and non-lymphoid cells	2.16E-19	106	12,576
M17411..	JAK-STAT signaling pathway	8.67E-18	133	16,062
rctm0838..	PKB-mediated events	1.23E-17	29	4,164
rctm1011..	Regulation of beta-cell development	4.17E-16	33	4,228
rctm0214	Caspase-mediated cleavage of cytoskeletal proteins	6.74E-13	11	1,851
rctm1147..	Signaling by TGF-beta receptor complex	3.56E-12	87	11,845
rctm0709	Mitochondrial protein import	3.80E-12	44	4,815
M12039	One carbon pool by folate	6.70E-12	17	2,834
M1467..	The co-stimulatory signal during T-cell activation	7.82E-12	35	4,239
rctm0495	Generic transcription pathway	4.21E-11	427	47,491
M8104..	Amino sugar and nucleotide sugar metabolism	5.68E-11	82	11,469
M17400..	ALK in cardiac myocytes	5.77E-11	42	6,555
rctm0540..	Homologous recombination repair	6.91E-11	16	2,604
M9131..	Glycerophospholipid metabolism	8.52E-11	91	13,344
M14449	METS effect on macrophage differentiation	1.09E-10	18	2,457
rctm0226	Cell-extracellular matrix interactions	2.01E-10	13	2,621
M7772	fl-arrestins in GPCR desensitization	2.96E-10	10	2,240
M9052	Phosphatidylinositol signaling system	1.07E-09	70	14,778
rctm0410	Facilitative Na+-independent glucose transporters	3.30E-09	12	1,986
M5244	Glyoxylate and dicarboxylate metabolism	3.98E-09	16	2,236
rctm0798	Nuclear receptor transcription pathway	5.98E-09	48	9,174
M13917	Role of Parkin in the ubiquitin-proteasomal pathway	1.57E-08	11	3,743
rctm0368	Elastic fibre formation	6.42E-08	41	6,860
rctm0602..	Integrin cell surface interactions	2.22E-07	102	20,947
rctm1250	Tandem pore domain potassium channels	2.56E-07	12	2,233
M2164	Leukocyte transendothelial migration	5.26E-07	107	20,207
rctm0967	Rap1 signaling	6.52E-07	15	3,892
rctm1220	Synthesis of glycosylphosphatidylinositol (GPI)	1.10E-06	15	1,766
M4047..	Selective expression of chemokine receptors during T-cell polarization	1.10E-06	44	6,205
rctm0891..	Potassium channels	1.59E-06	58	11,583

Table S1 (Continued)

Module* (n=103)	Description	FDR < 0.05	n of genes	n of SNPs
AD_Positive	Positive control for AD	2.25E-06	140	40,759
rctm0987..	Regulation of cholesterol biosynthesis by SREBP	3.44E-06	49	7,165
rctm1346..	Unfolded protein response	3.62E-06	59	6,852
M17906..	Vibrio cholerae infection	4.85E-06	76	13,174
rctm0662	MHC class II antigen presentation	9.85E-06	83	10,974
rctm0457	G0 and early G1	1.28E-05	24	2,793
M13883..	How progesterone initiates oocyte membrane	1.81E-05	75	11,619
rctm0933..	Pyruvate metabolism and the citric acid (TCA) cycle	2.29E-05	33	4,201
M6981	Protein export	2.79E-05	22	2,820
M4835	B cell survival pathway	6.04E-05	14	1,953
M4383..	Actions of nitric oxide in the heart	0.0001	58	12,815
rctm0647..	Lipid digestion, mobilization, and transport	0.0002	72	13,080
M10547	Role of MAL in rho-mediated activation of SRF	0.0002	19	2,421
M13950	Asthma	0.0003	15	1,562
rctm1201	Synthesis of PC	0.0004	17	2,839
rctm0054	Activation of Chaperones by ATF6-alpha	0.0004	11	1,349
rctm0809..	Opioid signaling	0.0005	327	53,980
rctm1178	Striated muscle contraction	0.0006	31	3,870
rctm0533..	Hexose transport	0.0007	46	6,105
rctm0174	Biotin transport and metabolism	0.0010	10	2,044
M2404	Mechanism of gene regulation by peroxisome proliferators via PPARa(alpha)	0.0010	54	8,543
rctm0854..	Peptide hormone metabolism	0.0011	53	7,739
rctm0221..	Cell death signaling via NRAGE, NRIF and NADE	0.0012	62	13,850
M3214	Primary bile acid biosynthesis	0.0012	16	1,903
rctm0547	Hyaluronan metabolism	0.0013	13	1,770
rctm0517	Golgi cisternae pericentriolar stack reorganization	0.0013	12	1,403
M1296	IL-7 signal transduction	0.0014	16	2,285
M17636..	Renin-angiotensin system	0.0017	20	2,734
rctm0714..	Mitotic anaphase	0.0020	97	13,551
rctm0118..	Antigen processing: ubiquitination and proteasome degradation	0.0022	200	28,927
rctm0678..	Membrane trafficking	0.0026	128	16,818
M8560..	Cell cycle: G2/M checkpoint	0.0033	35	4,786
rctm0817	Other semaphorin interactions	0.0033	18	4,264
rctm1091	Scavenging by Class A receptors	0.0034	18	3,750
M12851	Cell-to-Cell adhesion signaling	0.0036	14	5,405
rctm0271..	Constitutive signaling by NOTCH1 PEST domain mutants	0.0036	76	11,561
rctm0894	Pre-NOTCH processing in Golgi	0.0044	18	2,843
M15569	NOD-like receptor signaling pathway	0.0049	54	6,527

Table S1 (Continued)

**Module* (n=103)**	**Description**	**FDR < 0.05**	**n of genes**	**n of SNPs**
M6856	Hematopoietic cell lineage	0.0057	78	10,360
M12899	Glycosphingolipid biosynthesis - globo series	0.0061	13	1,986
M194	Proteasome complex	0.0062	17	1,966
rctm1040..	Resolution of AP sites via the single-nucleotide replacement pathway	0.0066	12	1,281
rctm0173	Biosynthesis of the N-glycan precursor (dolichol lipid-linked oligosaccharide [LLO]) and transfer to a nascent protein	0.0076	28	3,040
rctm0258..	Collagen biosynthesis and modifying enzymes	0.0085	65	14,557
M1547..	Control of skeletal myogenesis by HDAC and calcium/calmodulin-dependent kinase (CaMK)	0.0091	39	6,438
M2499	CARM1 and regulation of the estrogen receptor	0.0101	32	5,837
M3812	Amyotrophic lateral sclerosis (ALS)	0.0102	50	8,090
M16120	How Salmonella hijacks a cell	0.0103	12	1,626
M6929	*O*-glycan biosynthesis	0.0119	28	7,515
M917	Complement pathway	0.0121	15	2,415
rctm1336	Tryptophan catabolism	0.0121	11	1,742
M11358	Tumor suppressor Arf inhibits ribosomal biogenesis	0.0121	17	2,356
M5109	Pyrimidine metabolism	0.0122	91	12,987
rctm1109	Signal attenuation	0.0123	13	2,287
rctm0365	Effects of PIP2 hydrolysis	0.0125	24	6,850
rctm0418..	Fcgamma receptor (FcgR) dependent phagocytosis	0.0213	72	11,921
rctm0227..	Cellular response to hypoxia	0.0215	24	2,479
M5669	Natural killer cell mediated cytotoxicity	0.0261	121	17,356
M4891	Regulation of transcriptional activity by PML	0.0277	17	2,623
rctm1123..	Signaling by FGFR1 mutants	0.0278	29	4,765
rctm0960	RNA polymerase III transcription initiation	0.0283	35	4,104
rctm0619	Interleukin-6 signaling	0.0323	11	1,788
rctm1170..	Sphingolipid metabolism	0.0328	56	10,313
M12975	VEGF, hypoxia, and angiogenesis	0.0348	27	4,885
rctm0890..	Postsynaptic nicotinic acetylcholine receptors	0.0370	14	1,397
rctm1265	Canonical retinoid cycle in rods (twilight vision)	0.0386	16	2,042

CRP, C-reactive protein; FDR, false discovery rate; MSEA, marker-set enrichment analysis; SNP, single-nucleotide polymorphism.

* Modules marked with two periods (..) are those that are merged.

Table S2. MSEA analysis of CRP pathways (CRP, eQTL-based mapping; pathways arranged by ascending FDR)

**Module* (n=71)**	**Description**	**FDR < 0.05**	**n of genes**	**n of SNPs**
rctm0696..	Metabolism of vitamins and cofactors	4.54E-167	44	2,969
M12039	One carbon pool by folate	2.51E-86	10	1,267
M4319..	IL12 and Stat4 dependent signaling pathway in Th1 development	1.48E-71	19	1,110
rctm0495	Generic transcription pathway	1.98E-61	222	12,247
rctm0732..	MyD88 cascade initiated on plasma membrane	1.26E-55	61	3,010
rctm0798	Nuclear receptor transcription pathway	3.55E-54	13	417
rctm0166	Bile acid and bile salt metabolism	1.78E-52	10	699
M4741	Systemic lupus erythematosus	1.19E-50	43	1,800
rctm0458..	G1 phase	2.02E-49	30	1,743
rctm1155..	Signaling by NGF	2.14E-43	106	5,342
M14933..	Steroid hormone biosynthesis	2.85E-34	20	1,943
M5109..	Pyrimidine metabolism	1.93E-33	78	5,137
AD_Positive	Positive control for AD	1.19E-28	71	5,831
M4629	Nitrogen metabolism	9.50E-28	10	796
M9809..	Cytokine-cytokine receptor interaction	2.32E-27	117	4,413
M7330	Glycosaminoglycan biosynthesis - heparan sulfate	1.41E-21	15	501
M5785	Taste transduction	4.71E-21	10	648
M17758	Alanine, aspartate and glutamate metabolism	1.53E-19	15	1,200
rctm0602..	Integrin cell surface interactions	1.05E-17	42	2,352
M6856	Hematopoietic cell lineage	1.36E-17	31	1,279
rctm1133..	Signaling by NOTCH	6.04E-17	43	2,467
rctm0457	G0 and Early G1	7.58E-17	10	509
M766	Glycine, serine, and threonine metabolism	4.11E-16	14	1,123
rctm0517	Golgi cisternae pericentriolar stack reorganization	3.86E-14	11	606
M17857	SNARE interactions in vesicular transport	4.97E-13	23	1,730
rctm0291..	Cytosolic sensors of pathogen-associated DNA	6.32E-13	39	1,303
rctm0171..	Biological oxidations	5.71E-12	60	3,848
rctm0309	Deadenylation of mRNA	5.84E-11	10	157
rctm0985..	Regulation of APC/C activators between G1/S and early anaphase	1.53E-10	182	11,752
rctm0744	NCAM1 interactions	1.81E-10	13	332
rctm1333	Transport of vitamins, nucleosides, and related molecules	7.22E-10	12	918

Table S2 (Continued)

**Module* (n=71)**	**Description**	**FDR < 0.05**	**n of genes**	**n of SNPs**
rctm0522..	HIV Infection	2.98E-09	76	3,975
rctm0238..	Chromosome maintenance	4.34E-09	64	4,302
M9703	Role of BRCA1, BRCA2, and ATR in cancer susceptibility	5.54E-09	10	504
rctm1346..	Unfolded protein response	6.20E-09	42	1,517
rctm0654	Lysosome vesicle biogenesis	1.25E-08	13	517
M6981	Protein export	3.57E-08	12	527
rctm1229..	TGF-beta receptor signaling activates SMADs	3.87E-08	15	491
rctm0709	Mitochondrial protein import	8.94E-08	20	1,006
rctm0678	Membrane trafficking	9.13E-08	88	4,377
rctm0854..	Peptide hormone metabolism	2.34E-07	25	1,229
rctm0591..	Innate immune system	2.51E-07	110	4,381
rctm0388..	Extracellular matrix organization	5.10E-07	88	4,566
M17400..	ALK in cardiac myocytes	9.24E-07	20	633
M11911..	ABC transporters	2.72E-06	22	1,430
M6370	p53 signaling pathway	3.33E-06	31	2,397
rctm0684..	Metabolism of carbohydrates	3.42E-06	78	4,038
M4383	Actions of nitric oxide in the heart	2.25E-05	11	182
M1547..	Control of skeletal myogenesis by HDAC and calcium/calmodulin-dependent kinase (CaMK)	2.56E-05	17	340
rctm0775	Netrin-1 signaling	4.99E-05	18	788
rctm1169	Sphingolipid de novo biosynthesis	5.50E-05	15	542
M1519	Endocytosis	7.48E-05	90	4,076
rctm0339	Double-strand break repair	9.80E-05	13	655
rctm0598..	Integration of energy metabolism	0.0002	94	3,926
M8104	Amino sugar and nucleotide sugar metabolism	0.0009	30	2,117
rctm0933..	Pyruvate metabolism and the citric acid (TCA) cycle	0.0010	25	1,693
rctm1081	SLC-mediated transmembrane transport	0.0018	102	5,420
rctm0244..	Class B/2 (secretin family receptors)	0.0020	34	1,222
rctm0647	Lipid digestion, mobilization, and transport	0.0020	17	573
rctm0289..	Cytokine signaling in immune system	0.0041	49	1,636
rctm0115	Antigen activates B-cell receptor leading to generation of second messengers	0.0043	20	909
M615	Intestinal immune network for IgA production	0.0050	16	402
M11673	Biosynthesis of unsaturated fatty acids	0.0054	14	764

Table S2 (Continued)

**Module* (n=71)**	**Description**	**FDR < 0.05**	**n of genes**	**n of SNPs**
rctm0800	Nuclear signaling by ERBB4	0.0100	13	861
rctm0475..	GPCR downstream signaling	0.0121	159	7,055
M19895	Nicotinate and nicotinamide metabolism	0.0133	14	1,077
rctm1110	Signal regulatory protein (SIRP) family interactions	0.0193	11	478
M8601	Rac 1 cell motility signaling pathway	0.0195	13	745
M11106	Inactivation of Gsk3 by AKT causes accumulation of b-catenin in alveolar macrophages	0.0258	13	483
rctm1046..	Respiratory electron transport, ATP synthesis by chemiosmotic coupling, and heat production by uncoupling proteins.	0.0292	45	2,536
M9052..	Phosphatidylinositol signaling system	0.0417	51	2,046

CRP, C-reactive protein; eQTL, expression quantitative trait loci; FDR, false discovery rate; MSEA, marker-set enrichment analysis; SNP, single-nucleotide polymorphism.

* Modules marked with two periods (..) are those that are merged.

Table S3. MSEA analysis of IL6 pathways (IL6, 50-kb distance–based mapping; pathways arranged by ascending FDR)

**Module* (n=114)**	**Description**	**FDR < 0.05**	**n of genes**	**n of SNPs**
M6889	Riboflavin metabolism	3.26E-45	14	2,062
M16743..	Tyrosine metabolism	3.47E-32	71	10,034
rctm0174	Biotin transport and metabolism	1.05E-31	10	2,020
M13917	Role of Parkin in the ubiquitin-proteasomal pathway	4.55E-29	11	3,525
M13720..	Lysine degradation	4.97E-24	53	7,945
rctm0933..	Pyruvate metabolism and the citric acid (TCA) cycle	2.51E-20	42	5,770
rctm0542..	Hormone ligand-binding receptors	2.12E-18	18	2,332
M11792..	Sonic hedgehog (Shh) pathway	3.22E-18	26	4,803
M16894..	Complement and coagulation cascades	4.48E-17	79	11,184
rctm0281..	Cyclin A/B1 associated events during G2/M transition	6.69E-16	16	1,863
M10911..	Cysteine and methionine metabolism	4.94E-13	39	5,280
M16848..	Epithelial cell signaling in *Helicobacter pylori* infection	1.14E-12	88	14,178
rctm0061..	Activation of IRF3/IRF7 mediated by TBK1/IKK epsilon	7.34E-12	41	5,143
rctm1368	Voltage-gated potassium channels	9.30E-12	42	8,706
rctm0418..	Fc gamma receptor (FcgR) dependent phagocytosis	1.33E-11	110	14,986
rctm0692..	Metabolism of porphyrins	3.31E-11	14	1,957
rctm0688..	Metabolism of nitric oxide	1.34E-10	23	3,198
rctm0804	Nucleotide-like (purinergic) receptors	2.51E-10	12	1,473
M6778..	IL10 anti-inflammatory signaling pathway	6.01E-10	37	5,399
M4741	Systemic lupus erythematosus	8.44E-10	62	7,386
rctm0854..	Peptide hormone metabolism	8.79E-10	79	11,442
M2044	Spliceosome	1.47E-09	114	12,267
rctm0731..	Muscle contraction	2.08E-09	48	6,966
M19895	Nicotinate and nicotinamide metabolism	9.58E-09	20	2,695
rctm0974	Recruitment of NuMA to mitotic centrosomes	1.42E-08	10	1,233
M7528	Pantothenate and CoA biosynthesis	1.52E-08	16	2,792
M1840..	Glutathione metabolism	1.53E-08	49	4,756
M8809	Phosphoinositides and their downstream targets.	1.58E-08	22	4,223
M4361	Proximal tubule bicarbonate reclamation	2.23E-08	22	2,737
rctm0104	Amine-derived hormones	3.07E-08	15	2,221
rctm0800	Nuclear signaling by ERBB4	8.79E-08	35	10,774
M6856	Hematopoietic cell lineage	1.13E-07	79	10,133
M12718	Deregulation of CDK5 in Alzheimers Disease	1.16E-07	10	1,546

Table S3 (Continued)

Module* (n=114)	Description	FDR < 0.05	n of genes	n of SNPs
M5109..	Pyrimidine metabolism	1.26E-07	131	18,190
M638	Adherens junction	1.39E-07	72	17,998
M11521..	Glycolysis/gluconeogenesis	1.52E-07	71	9,872
M12039	One carbon pool by folate	1.66E-07	17	2,782
M13143..	uCalpain and friends in cell spread	2.24E-07	24	6,677
M963..	RNA degradation	2.37E-07	64	7,753
M4377	Galactose metabolism	3.01E-07	25	3,870
rctm0003..	3'-UTR–mediated translational regulation	3.05E-07	105	11,873
M523	Thyroid cancer	4.60E-07	29	4,664
M4844..	Chemokine signaling pathway	4.76E-07	118	17,763
rctm0872..	Platelet activation, signaling, and aggregation	6.28E-07	84	14,144
M4085	Primary immunodeficiency	8.93E-07	31	3,806
rctm0073	Activation of Rac	1.20E-06	13	3,490
rctm0696..	Metabolism of vitamins and cofactors	1.23E-06	80	12,408
rctm0035..	Acetylcholine binding and downstream events	1.71E-06	14	1,607
rctm0709	Mitochondrial protein import	1.75E-06	43	4,749
rctm0514	Glycosphingolipid metabolism	1.93E-06	33	6,006
rctm1346..	Unfolded protein response	1.93E-06	74	8,801
rctm0292	Cytosolic sulfonation of small molecules	2.37E-06	17	2,105
rctm0178	Branched-chain amino acid catabolism	2.70E-06	16	2,495
rctm1333..	Transport of vitamins, nucleosides, and related molecules	3.07E-06	31	5,640
M16991..	Skeletal muscle hypertrophy regulated via AKT/mTOR pathway	3.32E-06	24	3,394
M19708	Type II diabetes mellitus	3.35E-06	45	8,932
rctm0689..	Metabolism of non-coding RNA	8.52E-06	48	5,765
M16004	Antigen processing and presentation	1.10E-05	58	5,099
rctm0540..	Homologous recombination repair	1.24E-05	16	2,690
M1519	Endocytosis	1.58E-05	164	28,535
rctm0567	Immunoregulatory interactions between lymphoid and non-lymphoid cells	2.40E-05	69	8,040
rctm1229..	TGF-beta receptor signaling activates SMADs	3.02E-05	29	3,868
rctm0773	Nephrin interactions	4.47E-05	22	5,486
rctm0308	DSCAM interactions	6.37E-05	11	4,503
rctm0871..	Platelet aggregation (plug formation)	6.49E-05	37	5,745
M13088	PPAR signaling pathway	0.0002	66	9,463

Table S3 (Continued)

**Module* (n=114)**	**Description**	**FDR < 0.05**	**n of genes**	**n of SNPs**
rctm0873	Platelet calcium homeostasis	0.0002	18	4,505
rctm0573..	Inflammasomes	0.0003	17	2,478
rctm0494	Generation of second messenger molecules	0.0004	19	2,408
rctm0252..	Cleavage of growing transcript in the termination region	0.0005	29	3,572
rctm0624..	Ion channel transport	0.0006	101	21,502
M7963..	Cell cycle	0.0011	136	17,300
rctm0695..	Metabolism of steroid hormones and vitamin D	0.0016	28	4,145
rctm1287	Trafficking and processing of endosomal TLR	0.0021	11	1,386
M3952	Cells and molecules involved in local acute inflammatory response	0.0025	17	2,791
M1296..	IL7 signal transduction	0.0025	19	2,594
rctm0805..	*O*-linked glycosylation of mucins	0.0032	55	11,504
rctm1129	Signaling by Hippo	0.0037	17	3,112
rctm0870	Platelet adhesion to exposed collagen	0.0038	11	2,123
M12899	Glycosphingolipid biosynthesis—globo series	0.0038	13	1,824
rctm0517	Golgi cisternae pericentriolar stack reorganization	0.0047	12	1,446
M6981	Protein export	0.0047	22	3,115
rctm0115	Antigen activates B-cell receptor leading to generation of second messengers	0.0048	32	6,364
rctm1327..	Transport of inorganic cations/anions and amino acids/oligopeptides	0.0053	158	24,857
rctm1117..	Signaling by ERBB4	0.0080	127	21,734
rctm0128..	Arachidonic acid metabolism	0.0084	74	8,647
M12617..	Type I diabetes mellitus	0.0098	28	4,869
M3896..	Inositol phosphate metabolism	0.0105	67	11,598
M225	Downregulated of MTA-3 in ER-negative breast tumors	0.0151	18	2,543
rctm0415..	Fatty acid, triacylglycerol, and ketone body metabolism	0.0165	100	16,308
M1547	Control of skeletal myogenesis by HDAC and calcium/calmodulin-dependent kinase (CaMK)	0.0170	28	4,664
M111..	Roles of fl-arrestin-dependent recruitment of Src kinases in GPCR signaling	0.0173	18	3,383
M11266	Lysosome	0.0178	117	16,985
rctm0893..	Pre-NOTCH expression and processing	0.0199	42	6,093
rctm0997	Regulation of insulin secretion by acetylcholine	0.0214	10	3,443
rctm0775	Netrin-1 signaling	0.0220	40	11,381

Table S3 (Continued)

Module* (n=114)	Description	FDR < 0.05	n of genes	n of SNPs
rctm0677	Meiotic synapsis	0.0227	39	5,677
rctm0118..	Antigen processing: ubiquitination and proteasome degradation	0.0228	163	24,495
M5374	The SARS-coronavirus life cycle	0.0229	10	1,177
rctm0762	Na+/Cl– dependent neurotransmitter transporters	0.0242	18	2,875
rctm0013	AKT phosphorylates targets in the cytosol	0.0288	13	1,794
rctm1014	Regulation of gene expression in beta cells	0.0292	19	2,428
rctm1268	The phototransduction cascade	0.0297	29	4,862
rctm0312..	Defensins	0.0320	36	2,776
rctm1132..	Signaling by NODAL	0.0329	19	2,353
rctm0368	Elastic fiber formation	0.0331	41	7,094
M13324	Hypoxia-inducible factor in the cardiovascular system	0.0334	13	1,350
rctm0271..	Constitutive signaling by NOTCH1 PEST domain mutants	0.0338	62	9,671
rctm1110	Signal regulatory protein (SIRP) family interactions	0.0370	13	2,294
rctm0968	Ras activation upon Ca2+ influx through NMDA receptor	0.0370	17	3,734
M6370	p53 signaling pathway	0.0383	66	8,483
M10401	Telomeres, telomerase, cellular aging, and immortality	0.0421	17	4,390
rctm0947..	RNA polymerase I, RNA polymerase III, and mitochondrial transcription	0.0424	102	13,022
M4629..	Nitrogen metabolism	0.0426	22	2,521

FDR, false discovery rate; IL6, interleukin-6; MSEA, marker-set enrichment analysis; SNP, single-nucleotide polymorphism.

* Modules marked with two periods (..) are those that are merged.

Table S4. MSEA analysis of IL6 pathways (IL6, eQTL-based mapping to genes; pathways arranged by ascending FDR)

**Module* (n=55)**	**Description**	**FDR < 0.05**	**n of genes**	**n of SNPs**
M2044..	Spliceosome	4.20E-60	75	2,796
rctm1147..	Signaling by TGF-beta receptor complex	2.61E-47	33	1,363
M16743..	Tyrosine metabolism	7.75E-42	37	2,591
M699..	Fatty acid metabolism	6.82E-36	56	4,504
M9387..	Vascular smooth muscle contraction	1.25E-33	73	3,768
rctm1133..	Signaling by NOTCH	2.32E-27	40	2,829
rctm0475..	GPCR downstream signaling	5.14E-24	153	6,934
M14091..	Olfactory transduction	1.87E-23	21	1,075
M4361..	Proximal tubule bicarbonate reclamation	1.46E-22	18	914
M1519	Endocytosis	1.54E-22	88	4,473
rctm0707	Mitochondrial fatty acid beta-oxidation	1.98E-22	11	881
rctm0647	Lipid digestion, mobilization, and transport	3.25E-20	15	602
M19428..	Wnt signaling pathway	1.48E-19	70	3,273
rctm0690..	Metabolism of nucleotides	1.86E-17	46	3,278
rctm0388..	Extracellular matrix organization	3.73E-16	68	3,102
rctm1327..	Transport of inorganic cations/anions and amino acids/oligopeptides	4.08E-15	40	1,775
rctm1117..	Signaling by ERBB4	5.00E-12	89	5,324
rctm0894	Pre-NOTCH processing in Golgi	1.47E-11	13	1,742
M3985	The citrate cycle (TCA cycle)	4.87E-11	11	844
rctm0805..	*O*-linked glycosylation of mucins	5.84E-11	34	1,335
M7528	Pantothenate and CoA biosynthesis	6.27E-11	10	613
M2642..	TGF-beta signaling pathway	2.73E-10	43	1,848
M11266	Lysosome	4.39E-10	84	6,814
M1001..	Rho cell motility signaling pathway	1.05E-09	34	1,824
rctm0332..	Diseases associated with visual transduction	3.80E-09	30	1,039
M18306..	Regulation of actin cytoskeleton	5.77E-09	81	4,147
rctm1180..	Sulfur amino acid metabolism	5.68E-08	23	1,234
M5410..	Arachidonic acid metabolism	1.60E-07	28	1,485
rctm0686	Metabolism of lipids and lipoproteins	5.67E-07	265	14,798
rctm0845	PTM: gamma carboxylation, hypusine formation and arylsulfatase activation	6.09E-07	13	806
rctm0709	Mitochondrial protein import	9.00E-07	20	1,252
rctm1309	Translocation of GLUT4 to the plasma membrane	1.12E-06	29	2,261
rctm0689..	Metabolism of non-coding RNA	3.15E-06	47	2,911

Table S4 (Continued)

**Module* (n=55)**	**Description**	**FDR < 0.05**	**n of genes**	**n of SNPs**
M7330	Glycosaminoglycan biosynthesis—heparan sulfate	3.41E-06	15	458
M11190	Dorsoventral axis formation	5.82E-06	14	2,230
M7934	Pyruvate metabolism	6.87E-06	22	1,515
rctm0223..	Cell-surface interactions at the vascular wall	7.45E-06	76	4,133
M17844	Other glycan degradation	7.63E-06	15	1,083
rctm0625	Ion transport by P-type ATPases	1.36E-05	21	816
M4047..	Selective expression of chemokine receptors during T-cell polarization	1.50E-05	19	1,108
M17411	JAK-STAT signaling pathway	1.70E-05	46	1,547
rctm0492..	Gastrin-CREB signaling pathway via PKC and MAPK	2.57E-05	115	5,799
rctm1170..	Sphingolipid metabolism	0.0002	39	2,274
M14691..	Aminoacyl-tRNA biosynthesis	0.0005	32	2,582
M5539	Axon guidance	0.0005	52	2,561
M5940	Endocytotic role of NDK, phosphins and dynamin	0.0009	14	532
M5500	Base excision repair	0.0025	17	452
rctm0884..	Post-elongation processing of intronless pre-mRNA	0.0032	11	606
rctm0376	Energy-dependent regulation of mTOR by LKB1-AMPK	0.0058	10	625
M11675	Homologous recombination	0.0077	12	954
rctm0508..	Glycerophospholipid biosynthesis	0.0082	42	1,961
rctm0613	Interferon gamma signaling	0.0283	26	970
rctm0224..	Cell–cell communication	0.0310	50	2,186
rctm0261..	Complement cascade	0.0384	48	2,266
rctm0732..	MyD88 cascade initiated on plasma membrane	0.0475	87	3,154

FDR, false discovery rate; IL6, interleukin-6; eQTL, expression quantitative trait loci; MSEA, marker-set enrichment analysis; SNP, single-nucleotide polymorphism.

* Modules marked with two periods (..) are those that are merged.

Table S5. Tissue-specific shared pathways and KD genes across BC development and CRP/IL6

**Pathway Description**	**Adipose**	**Liver**	**PPI**
Iron uptake and transport¶	N/A	N/A	*ATP6V1D**, *ATP6V0C**
Glucose metabolism¥	N/A	N/A	*PYGB*†
Immunoregulatory interactions between lymphoid and non-lymphoid cells¶	*CD3G**	N/A	N/A
Selective expression of chemokine receptors during T-cell polarization¶¥	*CD3D**, *CD3G**	CD3G*	*IL4**†, *JAK2**†, *IL2**
JAK-STAT signaling pathway¥	N/A	N/A	*JAK1*†, *JAK3*†, *TYK2*†, *IL4*†, *STAT6*†

BC, breast cancer; CRP, C-reactive protein; eQTL, expression quantitative trait loci; IL6, interleukin-6; JAK-STAT, Janus kinase-signal transducer and activator of transcription; KD, key driver; N/A, not available; PPI, protein-to-protein interaction.
¶ Pathway overlapping supersets from CRP/IL6 meta-analysis on distance mapping.
¥ Pathway overlapping supersets from CRP/IL6 meta-analysis on eQTL mapping.
* KD member gene overlapped between BC and CRP/IL6 in the shared pathway on distance mapping.
† KD member gene overlapped between BC and CRP/IL6 in shared pathways on eQTL mapping.
